# Supplementary material for: Causal Relationship between Aging and Anorexia Nervosa: A White-Matter-Microstructure-Mediated Mendelian Randomization Analysis
Source: Biomedicines. 2024 Aug 16;12(8):1874. doi: 10.3390/biomedicines12081874 (PMC11351342; doi:10.3390/biomedicines12081874)
Supplement: Supplementary file 1 [file biomedicines-12-01874-s001.zip › Supplementary Figure/Figure S9/Leaveoneout plot of MR analysis for ubm-b-1873 to AN.pdf]

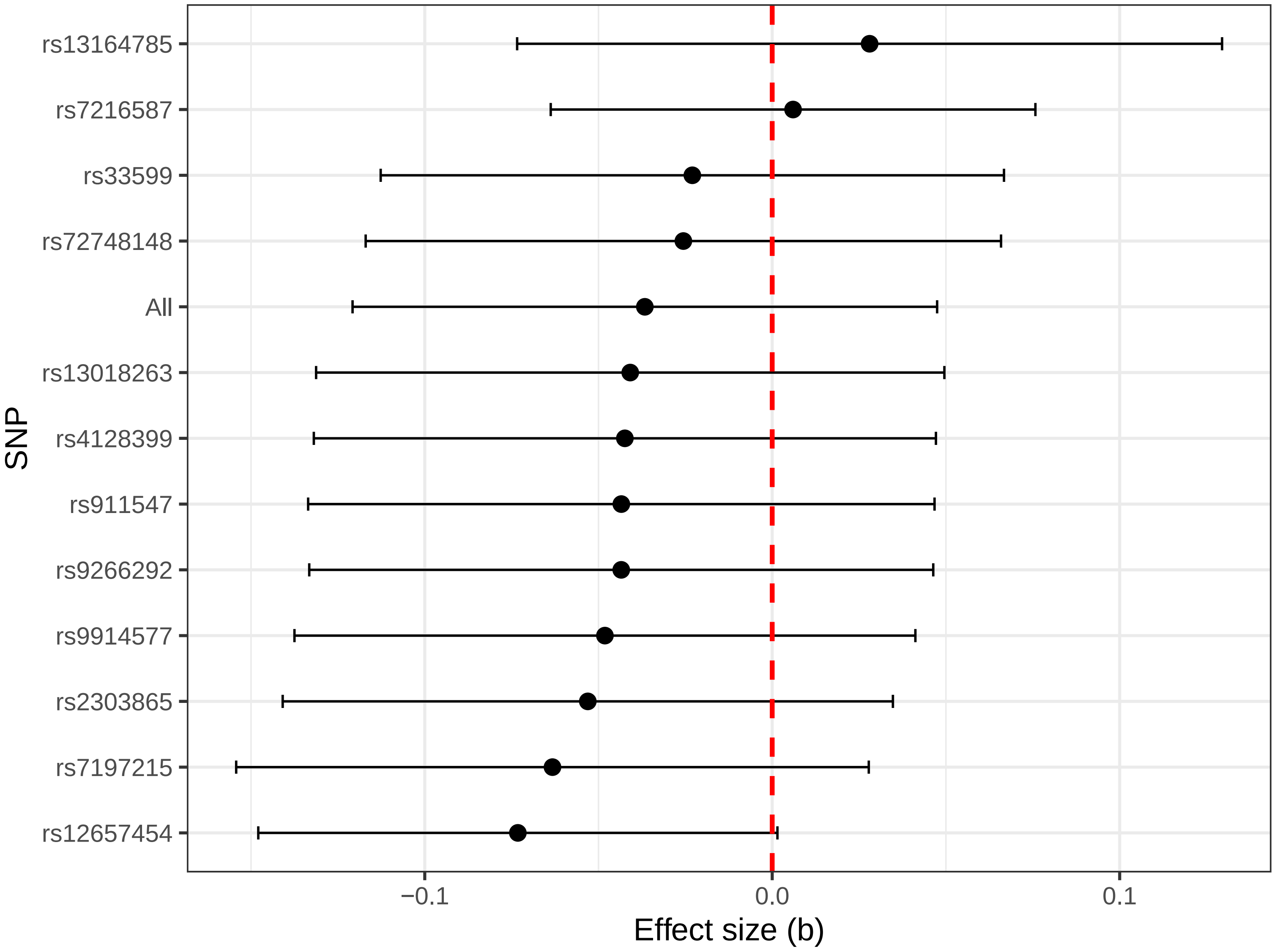
rs13164785
rs7216587
rs33599
rs72748148
All
rs13018263
rs4128399
rs911547
rs9266292
rs9914577
rs2303865
rs7197215
rs12657454
SNP
−0.1
0.0
0.1
Effect size (b)
